# The clinicopathological characteristics and genetic alterations between younger and older gastric cancer patients with curative surgery

**DOI:** 10.18632/aging.103627

**Published:** 2020-08-18

**Authors:** Chew-Wun Wu, Ming-Huang Chen, Kuo-Hung Huang, Shih-Ching Chang, Wen-Liang Fang, Chien-Hsing Lin, Yee Chao, Su-Shun Lo, Anna Fen-Yau Li, Yi-Ming Shyr

**Affiliations:** 1 Division of General Surgery, Department of Surgery, Taipei Veterans General Hospital, Taipei, Taiwan; 2 School of Medicine, National Yang-Ming University, Taipei, Taiwan; 3 Center of Immuno-Oncology, Department of Oncology, Taipei Veterans General Hospital, Taipei, Taiwan; 4 Division of Colon and Rectal Surgery, Department of Surgery, Taipei Veterans General Hospital, Taipei, Taiwan; 5 Genome Research Center, National Yang-Ming University, Taipei, Taiwan; 6 National Yang-Ming University Hospital, Yilan, Taiwan; 7 Department of Pathology, Taipei Veterans General Hospital, Taipei, Taiwan

**Keywords:** gastric cancer, age, clinicopathological feature, genetic alteration, prognosis

## Abstract

Few reports have investigated different genetic alterations according to age in various cancers. In total, 1749 GC patients receiving curative surgery were enrolled. The clinicopathological features, and prognoses were compared between younger (<65 years) and older (≥65 years) patients. Genetic mutations were analyzed using mass spectrometric single nucleotide polymorphism genotyping technology, including 68 validated mutations within eight genes (*TP53*, *ARID1A*, *BRAF*, and the *PI3K/AKT* pathway) previously reported in relation to age. Younger patients were more likely to be female and have poor cell differentiation, diffuse-type tumors, less lymphovascular invasion, fewer liver metastases, and better 5-year overall survival (OS) (68.0% vs. 54.6%, *P*<0.001) and disease-free survival (DFS) (65.4% vs. 53.0%, *P*<0.001) rates than older patients. Regarding the genetic alterations, older patients had more microsatellite instability-high (MSI-H) tumors and more *ARID1A* mutations than younger patients. Younger patients had significantly better OS and DFS rates than older patients for each pathological Tumor, Node, Metastasis (TNM) stage. Older patients had a significantly higher non-cancer related death rate than younger patients (36.2% vs. 12.3%, *P*<0.001). Age was an independent prognostic factor in GC. In conclusion, age was associated with different clinicopathological features and genetic alterations in GC with curative surgery.

## INTRODUCTION

Gastric cancer (GC) is the sixth most common cancer and the second most common cause of cancer-related deaths worldwide [1]. Surgical intervention remains the only curative treatment for GC. Individuals between 50 and 70 years old show the most frequent GC prevalence.

A large series comparing the clinicopathological and molecular features between younger (<45 years) and older (≥45 years) GC patients [2] demonstrated that younger patients were associated with a female predominance and a more advanced stage, less frequent *TP53* and *HER-2* overexpression, fewer microsatellite instability-high (MSI-H) tumors, a higher level of *Helicobacter pylori* (HP) infection, and worse cancer-specific survival than older patients. Younger patients had higher cancer-related mortality than older patients.

To the best of our knowledge, few reports have compared the genetic mutations between younger and older GC patients. The incidence of *TP53* mutations was reported to be different between younger and older GC patients [3]. In addition to GC, there is a lack of comprehensive and economic genetic analysis method for cancer-related genes that have been reported to differ between younger and older cancer patients in cancers related or non-related to the gastrointestinal tract. In colorectal cancer, loss of *ARID1A* expression was associated with a younger age [4]. In papillary thyroid carcinoma, patients with *BRAF* expression were associated with older age and higher tumor recurrence rates than patients without *BRAF* expression [5]. In endometrial carcinoma, *PIK3CA* amplifications, but not *PIK3CA* mutation, were associated with older age [6]. In breast cancer, *PTEN* mutations were associated with younger age [7].

According to the World Health Organization (WHO), most developed countries use a chronological age of 65 years or older to categorize elder or older persons. Therefore, in the present study, we separated patients into younger age (<65 years) and older age (≥65 years) groups. Since whole genome sequencing is expensive, we used mass spectrometric single nucleotide polymorphism genotyping technology for multiplex analysis, which included 68 validated mutations within eight genes (*TP53*, *ARID1A*, *BRAF*, and the *PI3K/AKT* pathway) [8] in accordance with previous data that mutations in these genes were shown for cancers related or non-related to the gastrointestinal tract. The aim of the present study was to compare the clinicopathological features, recurrence patterns, prognoses, and genetic alterations between younger and older GC patients with curative surgery.

## RESULTS

### Clinicopathological features

As shown in Table 1, younger patients (<65 years) were more likely to be female, and have poor cell differentiation, diffuse-type tumors, and less lymphovascular invasion than older patients (≥65 years).

**Table 1:** Clinical profile in GC patients in different age groups.

**Variables**	**Age <65 years n=618 n (%)**	**Age ≥65 years n=1131 n (%)**	***P* value**
Gender (M/F)	341/277	914/217	**<0.001**
Tumor size (<5/≥5 cm)	368/250	631/500	0.129
Cell differentiation			**<0.001**
Poor	430 (69.6)	450 (39.8)	
Moderate	183 (29.6)	657 (58.1)	
Well	5 (0.8)	24 (2.1)	
Gross appearance			**<0.001**
Superficial type	251 (40.6)	400 (35.4)	
Borrmann type 1 and 2	109 (17.6)	308 (27.2)	
Borrmann type 3 and 4	258 (41.7)	423 (37.4)	
Lauren’s classification			**<0.001**
Intestinal type	235 (38.0)	787 (69.6)	
Diffuse type	383 (62.0)	344 (30.4)	
Lymphovascular invasion	302 (48.9)	610 (53.9)	**0.043**
Pathological T category			0.090
T1/2/3/4	224/80/231/83	386/188/383/174	
Pathological N category			0.524
N0/1/2/3	281/91/95/151	552/145/170/264	
Pathological TNM Stage			0.447
I/II/III	243/152/223	471/250/410	

TNM: Tumor, Node, Metastasis; Bold: statistically significant

### Initial recurrence patterns

As shown in Table 2, among the 1749 patients, younger patients tended to have fewer liver metastases than older patients.

**Table 2:** The initial recurrence pattern in gastric cancer patients in different age groups.

	**Age <65 years n=618 n (%)**	**Age ≥65 years n=1131 n (%)**	***P* value**
Total patients with recurrence	119 (19.3)	212 (18.7)	0.794
Locoregional recurrence	51 (8.3)	84 (7.4)	0.536
Distant metastasis	94 (15.2)	179 (15.8)	0.734
Peritoneal dissemination	54 (8.7)	71 (6.3)	0.056
Hematogenous metastasis	39 (6.3)	100 (8.8)	0.061
Liver	21 (3.4)	73 (6.5)	**0.007**
Lung	7 (1.1)	18 (1.6)	0.440
Bone	10 (1.6)	12 (1.1)	0.318
Brain	3 (0.5)	1 (0.1)	0.097
Adrenal	1 (0.2)	3 (0.3)	0.665
Skin	2 (0.3)	2 (0.2)	0.539
Distant lymphatic recurrence	18 (2.9)	43 (3.8)	0.333

Some patients had more than one recurrence patter; Bold: statistically significant

### Survival analysis

**Figure 1:**
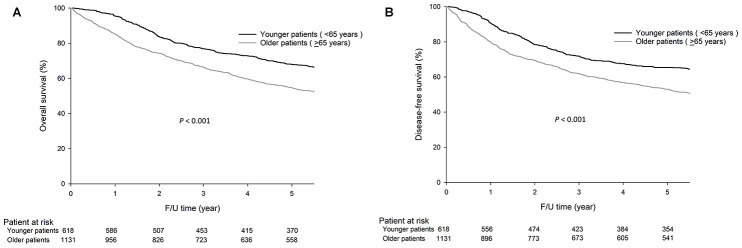
**The 5-year OS (68.0% vs. 54.6%, *P*<0.001) and DFS (65.4% vs. 53.0%, *P*<0.001) rates were significantly higher in younger patients (<65 years) than in older patients (≥65 years).** The survival curves are shown as follows: (**A**) OS curves of GC patients. (**B**) DFS curves of GC patients.

As shown in Figure 1, the 5-year overall survival (OS) (68.0% vs. 54.6%, *P*<0.001) and disease-free survival (DFS) (65.4% vs. 53.0%, *P*<0.001) rates were higher in younger patients than in older patients.

**Figure 2:**
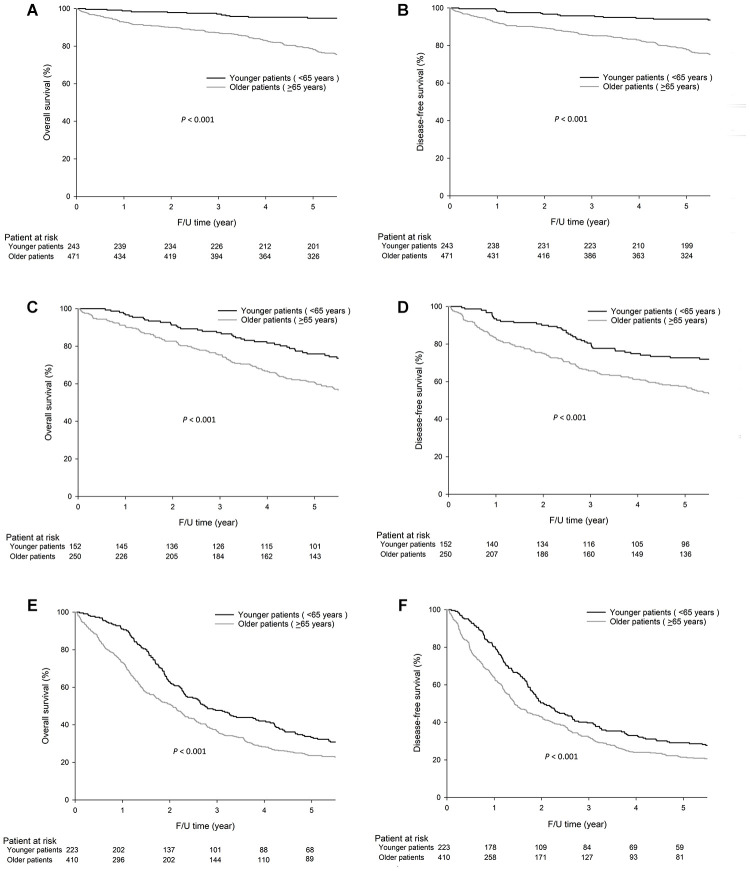
**For stage I GC, the 5-year OS (94.9% vs. 78.2%, *P*<0.001) and DFS (94.1% vs. 77.8%, *P*<0.001) rates were significantly higher in younger patients (< 65 years) than older patients (≥65 years), and similar results were observed for stage II GC, the 5-year OS, 75.9% vs. 60.6%, *P*<0.001 and DFS, 72.7% vs. 57.5%, *P*<0.001) and stage III GC, 5-year OS, 33.3% vs. 23.6%, *P*<0.001 and DFS, 29.1% vs. 21.4%, *P*=0.001.** The survival curves are shown as follows: (**A**) OS curves of stage I GC patients. (**B**) DFS curves of stage I GC patients. (**C**) OS curves of stage II GC patients. (**D**) DFS curves of stage II GC patients. (**E**) OS curves of stage III GC patients. (**F**) DFS curves of stage III GC patients.

As shown in Figure 2, the OS and DFS rates were significantly higher in younger patients than in older patients for stage I GC (5-year OS, 94.9% vs. 78.2%, *P*<0.001, Figure 2A and DFS, 94.1% vs. 77.8%, *P*<0.001, Figure 2B); stage II GC (5-year OS, 75.9% vs. 60.6%, *P*<0.001, Figure 2C and DFS, 72.7% vs. 57.5%, *P*<0.001, Figure 2D); and stage III GC (5-year OS, 33.3% vs. 23.6%, *P*<0.001, Figure 2E and DFS, 29.1% vs. 21.4%, *P*=0.001, Figure 2F).

The univariate analysis demonstrated that the following seven factors were associated with OS and DFS: age, gender, tumor size, cell differentiation, lymphovascular invasion, Lauren’s classification, and pathological Tumor, Node, Metastasis (TNM) stage (Table 3). The aforementioned seven variables were included in a multivariate Cox proportional hazards model to adjust for the effects of covariates. The multivariate analysis demonstrated that age, tumor size, and pathological TNM stage were independent prognostic factors affecting OS and DFS (Table 3).

**Table 3:** Univariate and multivariate analysis of factors affecting OS and DFS of GC patients after curative surgery.

**Prognostic factors**	**Univariate analysis**					
	**OS**			**DFS**		
	**HR**	**95%CI**	***P* value**	**HR**	**95%CI**	***P* value**
Age	1.46	1.135-1.878	**0.003**	1.40	1.095-1.795	**0.007**
Gender	0.72	0.546-0.948	**0.019**	0.71	0.543-0.938	**0.016**
Tumor size	2.44	1.850-3.227	**<0.001**	2.41	1.835-3.168	**<0.001**
Cell differentiation	0.76	0.600-0.963	**0.023**	0.75	0.596-0.951	**0.017**
Lymphovascular invasion	2.69	1.964-3.680	**<0.001**	2.57	1.890-3.485	**<0.001**
Lauren’s classification	1.33	1.044-1.689	**0.021**	1.32	1.044-1.680	**0.021**
Adjuvant chemotherapy	0.86	0.609-1.199	0.364	0.89	0.643-1.239	0.496
Pathological TNM stage	2.52	2.125-2.981	**<0.001**	2.33	1.985-2.735	**<0.001**
**Prognostic factors**	**Multivariate analysis**					
	**OS**			**DFS**		
	**HR**	**95%CI**	***P* value**	**HR**	**95%CI**	***P* value**
Age	1.47	1.126-1.920	**0.005**	1.39	1.071-1.808	**0.013**
Gender	1.11	0.827-1.491	0.485	1.07	0.799-1.436	0.646
Tumor size	1.43	1.066-1.929	**0.017**	1.46	1.086-1.951	**0.012**
Cell differentiation	0.89	0.614-1.300	0.556	0.87	0.604-1.255	0.458
Lymphovascular invasion	1.14	0.798-1.620	0.478	1.13	0.799-1.603	0.485
Lauren’s classification	1.08	0.740-1.586	0.680	1.06	0.728-1.540	0.765
Pathological TNM stage	2.29	1.868-2.809	**<0.001**	2.10	1.729-2.552	**<0.001**

CI: confidence interval; HR: hazard ratio; OS: overall survival; DFS: disease-free survival; TNM: Tumor, Node, Metastasis; Bold: statistically significant;

### Causes of death

Among the 1749 patients, 639 died before the last follow-up. The causes of death in younger patients included 28 (12.3%) non-cancer-related deaths, 190 (83.7%) GC-related deaths, and 9 (4.0%) other cancer-related deaths. The causes of death in older patients included 250 (36.2%) non-cancer-related deaths, 405 (58.7%) GC-related deaths, and 35 (5.1%) other cancer-related deaths. Older patients had significantly higher non-cancer-related death rates than younger patients (36.2% vs. 12.3%, *P*<0.001).

For stage I GC, older patients had a slightly higher non-cancer-related death rate than younger patients (60.7% vs. 42.9%, *P*=0.059). For stage II GC, older patients had a significantly higher non-cancer-related death rate than younger patients (39.7% vs. 10.6%, *P*=0.001), which was also observed in stage III GC (19.8% vs. 8.8%, *P*=0.006).

### Analysis of genetic alterations

As shown in Table 4, older patients had more MSI-H tumors and *ARID1A* mutations than younger patients. Genetic mutations in the *PI3K/AKT* pathway, *TP53*, and *BRAF* were not significantly different between the age groups.

**Table 4:** Comparison of the molecular differences between different age groups.

**Variables**	**Age <65 years n=185 n (%)**	**Age ≥65 years n=248 n (%)**	***P* value**
MSI status			**0.007**
MSI-H	9 (4.9)	31 (12.5)	
MSI-L/S	176 (95.1)	217 (87.5)	
HP infection	105 (56.8)	121 (48.8)	0.101
EBV infection	28 (15.1)	29 (11.7)	0.295
*PIK3CA* amplification	60 (32.4)	93 (37.5)	0.275
Genetic mutations			
*PI3K/AKT* pathway	21 (11.4)	36 (14.5)	0.335
*TP53*	20 (10.8)	27 (10.9)	0.980
*ARID1A*	9 (4.9)	27 (10.9)	**0.025**
*BRAF*	0	1 (0.4)	0.387

MSI: microsatellite instability; MSI-H: MSI-high; MSI-L/S: MSI-low/stable; HP: Helicobacter pylori; EBV: Epstein-Barr virus; Bold: statistically significant

Regarding the genetic mutations in the *PI3K/AKT* pathway, the frequencies of each mutated gene between younger and older patients were as follows: *PIK3CA* (7.6% vs. 9.7%, *P*=0.443), *PTEN* (3.2% vs. 4.4%, *P*=0.527), *AKT1* (0.5% vs. 0%, *P*=0.246), *AKT2* (0% vs. 0.4%, *P*=0.387), *AKT3* (0% vs. 0.8%, *P*=0.221), respectively.

### Analysis of the differences in clinicopathological features, patient prognosis, and genetic alterations between three different age groups

Because individuals between 50 and 70 years old show the most frequent GC prevalence, patients between 50 and 70 years may share the same disease characteristics, pathogenesis and clinicopathological characteristics. In addition, age is better appreciated as a continuous variable and variation exists in individuals of the same age, and any predefined age cut-off is an arbitrary rather than an unequivocal definition, however, several reasons led majority individual studies and international groups to define patients younger than 40 years old as younger patients [9]. Moreover, the number of patients younger than 40 years old is only 57 in the present study, showing that 40 years old is not appropriate to be the cut-off point of age due to disparity. According to the above reasons, we separate the enrolled patients into three age groups: group 1: age <50 years; group 2: age 50-70 years; and group 3: age >70 years.

As shown in Supplementary Table 1, group 1 was more likely to be female and have poor cell differentiation, superficial-type tumors, diffuse-type tumors, and less lymphovascular invasion than groups 2 and 3. Regarding the initial recurrence patterns, as shown in Supplementary Table 2, among the 1749 patients, those in group 1 tended to have fewer hematogenous and liver metastases than those in groups 2 and 3.

As shown in Supplementary Figure 1, the 5-year OS rates (74.4% vs. 63.3% vs. 53.3%, *P*<0.001) and DFS rates (70.8% vs. 61.3% vs. 51.7%, *P*<0.001) were higher in group 1 than in groups 2 and 3. As shown in Supplementary Figure 2A, for stage I GC, the 5-year OS (94.1% vs. 93.2% vs. 76.1%, *P*<0.001) and DFS (93.0% vs. 92.9% vs. 75.6%, *P*<0.001) rates were significantly higher in groups 1 and 2 than in group 3. For stage II GC (Supplementary Figure 2B), the 5-year OS (80.1% vs. 72.8% vs. 57.8%, *P*<0.001) and DFS (77.1% vs. 68.5% vs. 55.4%, *P*<0.001) rates were significantly higher in group 1 than in groups 2 and 3. For stage III GC, the 5-year OS (43.9% vs. 25.6% vs. 24.5%, *P*<0.001) and DFS (36.7% vs. 23.3% vs. 22.1%, *P*=0.003) rates were significantly higher in group 1 than in groups 2 and 3.

The univariate analysis demonstrated that the following seven factors were associated with OS and DFS: age, gender, tumor size, cell differentiation, lymphovascular invasion, Lauren’s classification, and pathological TNM stage. The aforementioned seven variables were included in a multivariate Cox proportional hazards model to adjust for the effects of covariates. The multivariate analysis demonstrated that age, gender, tumor size, lymphovascular invasion, and pathological TNM stage were independent prognostic factors affecting OS and DFS (Supplementary Table 3).

As shown in Supplementary Table 4, group 3 had more MSI-H tumors (group 1: 1.6%, group 2: 9.6%, group 3: 12.6%, respectively, *P*=0.038) and more *ARID1A* mutations (group 1: 3.2%, group 2: 7.4%, group 3: 11.0%, respectively, *P*=0.044) than groups 1 and 2. The genetic mutations in the *PI3K/AKT* pathway, *TP53*, and *BRAF* were not significantly different between different age groups. Regarding the genetic mutations in the *PI3K/AKT* pathway, the frequencies of each mutated gene between groups 1, 2, and 3 were as follows: *PIK3CA* (11.1% vs. 7.4% vs. 9.3%, *P*=0.632), *PTEN* (4.8% vs. 2.1% vs. 5.5%, *P*=0.411), *AKT1* (0% vs. 0.5% vs. 0%, *P*=0.695), *AKT2* (0% vs. 0.5% vs. 0%, *P*=0.695), *AKT3* (0% vs. 0.5% vs. 0.5%, *P*=0.649), respectively.

## DISCUSSION

For GC patients with curative surgery, our results showed that older patients had worse survival outcomes than younger patients; similar results were also obtained at each TNM stage. Age itself is an independent prognostic factor. Older patients had more liver metastasis than younger patients. Regarding the molecular analysis, older patients had more MSI-H tumors and *ARID1A* mutations than younger patients. Our novel findings demonstrated that the modular differences varied between younger and older patients.

A large series demonstrated that young-onset GC patients had more advanced disease than older-onset GC patients, and age was not an independent prognostic factor [2]. However, in the present study, tumor stage was not significantly different between younger and older patients for all enrolled patients. Younger patients might have had better survival outcomes than older patients because they had relatively better health conditions and were more willing to undergo and could better tolerate adjuvant chemotherapy. The percentage of GC patients receiving adjuvant chemotherapy was significantly higher among younger patients (18.9%) than older patients (8.4%). The low percentage of adjuvant chemotherapy in our patient population occurred because adjuvant chemotherapy was not routinely performed before 2008, after which time it was shown to have a survival benefit [10]. Although adjuvant chemotherapy was not associated with patient survival in the present study, the increase in the initial recurrence pattern in older patients may be associated with less use of chemotherapy than in younger patients. In addition, younger patients might have received more aggressive chemotherapy than older patients; however, this hypothesis was not tested because the detailed chemotherapy regimens for each patient were not available in our database. In the present study, older patients had a significantly higher non-cancer-related death rate than younger patients (36.2% vs. 12.3%, *P*<0.001), which was similar to the findings of Seo et al [2]. Consequently, another reason why older patients had a worse survival rate than younger patients may be due to the increased amount of non-cancer-related deaths among older patients.

For clinical use and economic concerns, we used mass spectrometric SNP genotyping technology for mutational analysis in the present study. However, integrating multiplatform genomic features is currently quite popular for cancer studies. Vertical integration of multidimensional omics data is indispensable to determine a subset of important prognostic features, such as cancer phenotype, cancer status, and patient survival [11]. Our future study will focus on integrating multiplatform genomic analysis, and we believe that integration studies on multi-omics data will provide insights into investigating novel biomarkers for cancer treatment.

Our results showed no significant difference in *TP53* mutations between younger and older patients, which was inconsistent with the findings of Rahman et al [3], who demonstrated more *TP53* mutations in younger patients than older patients. Rahman et al reported a relatively higher incidence of HP infection (51/71, 71.8%) and *TP53* mutation (52/71, 73.2%) in their patient group than the present study, and a significant correlation between HP infection and *TP53* mutation was observed. In their study, 63.4% of patients were smokers, and 75% of these patients had *TP53* mutations. In addition, some patients had a routine habit of consuming excess salt in their daily meals. A high salt diet and HP infection have been associated with the development of GC [12, 13]. Furthermore, smoking was reported to be associated with *TP53* mutation [14]. The incidence of *TP53* mutation in patients younger than 40 years was 94.4% [3], while no patients with *TP53* mutation were observed among patients in our study who were younger than 40 years. The discrepancy in *TP53* mutations between the present study and the study by Rahman et al [3] might be due to differences in the incidence of HP infection and the smoking and eating habits, which are considered to be the environmental factors.

The novel finding of the present study is that older patients had more *ARID1A* mutations than younger patients, which has not previously been reported. Even though we divided the patients into three age groups, the frequency of *ARID1A* mutations increased significantly with age. In colorectal cancer, loss of *ARID1A* expression was associated with younger age [4]. *ARID1A* is identified as a tumor suppressor gene in various cancers, especially gynecologic cancers, and an inverse correlation between *ARID1A* expression and tumor stage has been reported [15–17]. In the present study, patients with *ARID1A* mutations had significantly more MSI-H tumors than patients without *ARID1A* mutations (25% vs. 7.8%, *P*=0.001), which was similar to the results of other studies [18]. As a result, our older patients had more MSI-H tumors than younger patients, which may lead to a higher frequency of *ARID1A* mutations in older patients.

Environmental factors such as HP infection and dietary and smoking habits are known to impact the histologic type and genetic alterations during gastric carcinogenesis. Patients with HP infection were more likely to have *KRAS* mutation than patients without HP infection [19]. Another environmental factor, such as EBV infection, was reported to be associated with genetic mutations (*AKT2*, *CCNA1*, *MAP3K4*, and *TGFBR1*) and hypermethylation (*ACSS1*, *FAM3B*, *IHH*, and *TRABD*) in GC tissues; *AKT2* mutation was associated with a poor survival in GC patients with EBV infection [20]. Age might be treated as an environmental factor that is correlated with the gene-environment interactions, resulting in an association between age and clinicopathological and molecular features.

There are some limitations in the present study. First, this is a retrospective study and selection bias exists. Second, not all enrolled patients had available tumor samples for the analysis of genetic alterations. Third, younger patients with more advanced disease and older patients with poor physical conditions are all not candidates for surgery, excluding these patients may severely biased the assessment of effect of age on GC patients. Although the authors only want to study on patients with curative GC, these patients were firstly selected based on conditions suitable for surgery, age is less important in these patients than in all GC patients, which can help to guide prevention, prediction, prognosis and treatment. Forth, this study included patients hospitalized from 1998-2013, in which period the treatments of GC, such as adjuvant therapy, perioperative therapy, D2 gastrectomy, minimally invasive surgery, endoscopic therapy and targeted therapy, all are inconsistent between the patients and may affect prognosis. Fifth, according to The Cancer Genome Atlas (TCGA) dataset [21], the mutation frequencies of *AKT1* (0.9%), *AKT2* (2.3%), *AKT3* (1.8%) and *BRAF* (3.0%) are very low. Although we don't know which of them are driver genes and which of them are passenger genes, the test power is too weak to make a meaningful outcome when to compare their differences.

In conclusion, age was associated with clinicopathological features, recurrence patterns, patient prognoses and genetic alterations in GC with curative surgery. Our findings might be advanced by investigating gene-environment interactions while treating age as an environmental factor.

## MATERIALS AND METHODS

### Patients and sample collection

A total of 1749 patients who underwent curative surgery for gastric adenocarcinoma between 1998 and 2013 were enrolled. Patients who had gastric stump cancer or a history of previous gastric surgery were excluded.

A total of 433 patients with available tissues were enrolled to analyze genetic alterations. Tumor tissues and normal gastric mucosa tissues were collected and stored in a biobank at our institution. The study was approved by the Ethical Committee of Taipei Veterans General Hospital and was performed in accordance with the Declaration of Helsinki. Written informed consent was obtained from all study participants. The pathological staging of GC was performed according to the 8^th^ American Joint Committee on Cancer (AJCC)/Union for International Cancer Control (UICC) TNM classification system [22].

### Follow-up

Follow-up examinations were performed every 3 months during the first 3 years after surgery and every 6 months thereafter and included physical examinations, blood tests with measurements of tumor markers, chest radiography, and sonography or computerized tomography scans of the abdomen.

Adjuvant chemotherapy after curative surgery was not routinely performed in our institute prior to 2008; these treatments were applied only when tumor recurrence was diagnosed or highly suspected. TS-1 adjuvant chemotherapy was started for stage II and III patients in 2008 due to its proven survival benefits [9].

### DNA extraction

DNA extraction from tissue specimens was performed using the QIAamp DNA Tissue Kit and MinElute Virus Kit (Qiagen, Valencia, CA) according to a previous report [8].

### Detection of HP and Epstein-Barr virus (EBV) infection

Both tumor and nontumor tissues were assessed for HP infection with the polymerase chain reaction (PCR) method. The sequence of the HP reference genome (GenBank: AE000511.1) was used recording to a previous report [23].

EBV DNA assays were carried out using the Sequenom MassARRAY system (Sequenom, San Diego, CA) according to a previous report [23].

### MSI analysis

As described in a previous study [24], five reference microsatellite markers, namely, D5S345, D2S123, D17S250, BAT25 and BAT26, were used to determine the presence of MSI. MSI-H was defined as ≥ 2 loci of instability with 5 markers, while MSI-low/stable (MSI-L/S) was defined as one locus or no MSI loci.

### MassARRAY-based mutation characterization

A MassARRAY system (Agena, San Diego, CA) was used to identify 68 mutation hotspots in 8 GC-related genes (*TP53*, *ARID1A*, *PTEN*, *PIK3CA*, *AKT1*, *AKT2*, *AKT3*, and *BRAF*) [8]. *PI3K/AKT* pathway genetic mutations were defined as mutations identified in *PIK3CA*, *PTEN*, *AKT1*, *AKT2*, or *AKT3*.

### Statistical analysis

IBM SPSS Statistics 25.0 was used for statistical analyses. A χ^2^ test with Yates correction or Fisher’s exact test was used to compare categorical data. OS was defined from the date of surgery to the date of death or the last follow-up, while DFS was defined as the length of time after surgery during which the patient was alive without GC recurrence. The Kaplan–Meier method was used to perform the survival analysis and draw survival curves of OS and DFS. A multivariate analysis with Cox proportional hazards models was performed to analyze the independent prognostic factors of OS and DFS. A *P* value <0.05 was defined as statistically significant.

## Supplementary Material

Supplementary Figures

Supplementary Tables
