# Supplementary material for: The clinicopathological characteristics and genetic alterations between younger and older gastric cancer patients with curative surgery
Source: Aging (Albany NY). 2020 Aug 18;12(18):18137–50. doi: 10.18632/aging.103627 (PMC7585087; doi:10.18632/aging.103627)
Supplement: Supplementary Figures [file aging-12-103627-s002..pdf]

SUPPLEMENTARY FIGURES

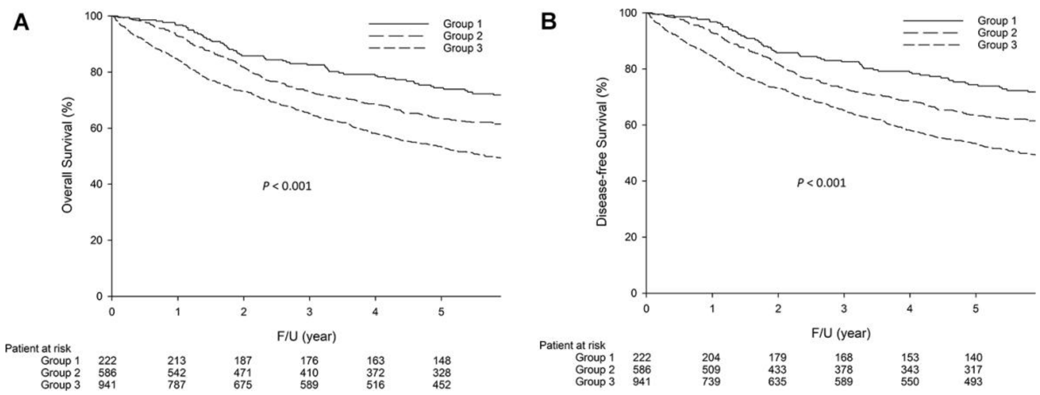

Supplementary Figure 1. The 5-year OS (74.4% vs. 63.3% vs. 53.3%, P<0.001) and DFS (70.8% vs. 61.3% vs. 51.7%, P<0.001) rates were significantly higher in group 1 than in groups 2 and 3. The survival curves are shown as follows: (**A**) OS curves of GC patients. (**B**) DFS curves of GC patients.

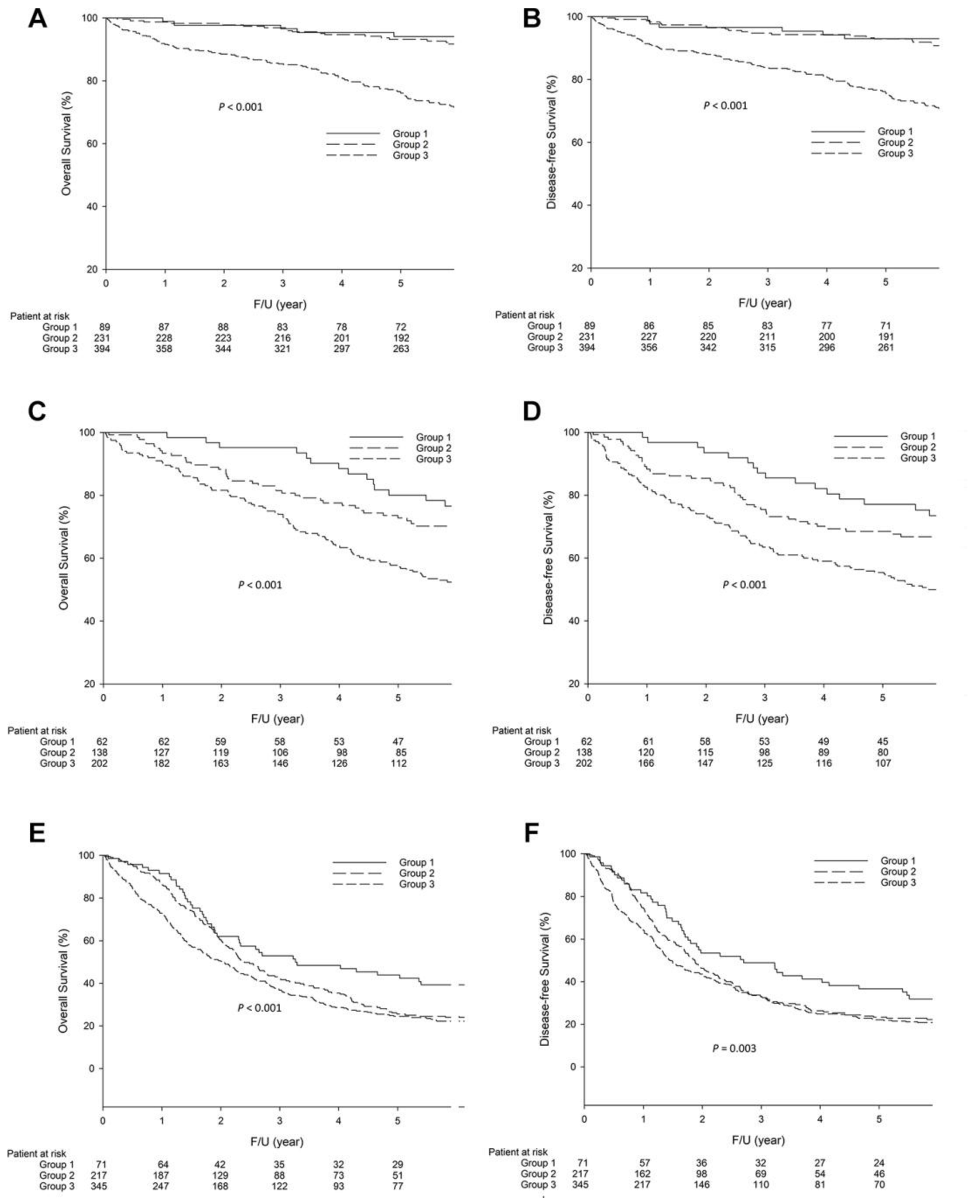

Supplementary Figure 2. For stage I GC, the 5-year OS (94.1% vs. 93.2% vs. 76.1%, P<0.001) and DFS (93.0% vs. 92.9% vs. 75.6%, P<0.001) rates were significantly higher in groups 1 and 2 than in group 3. For stage II GC, the 5-year OS (80.1% vs. 72.8% vs. 57.8%, P<0.001) and DFS (77.1% vs. 68.5% vs. 55.4%, P<0.001) rates were significantly higher in group 1 than in groups 2 and 3. For stage III GC, the 5-year OS (43.9% vs. 25.6% vs. 24.5%, P<0.001) and DFS (36.7% vs. 23.3% vs. 22.1%, P=0.003) rates were significantly higher in group 1 than in groups 2 and 3. The survival curves are shown as follows: (**A**) OS curves of stage I GC patients. (**B**) DFS curves of stage I GC patients. (**C**) OS curves of stage II GC patients. (**D**) DFS curves of stage II GC patients. (**E**) OS curves of stage III GC patients. (**F**) DFS curves of stage III GC patients.
