# Supplementary material for: The clinicopathological characteristics and genetic alterations between younger and older gastric cancer patients with curative surgery
Source: Aging (Albany NY). 2020 Aug 18;12(18):18137–50. doi: 10.18632/aging.103627 (PMC7585087; doi:10.18632/aging.103627)
Supplement: Supplementary Tables [file aging-12-103627-s001..pdf]

SUPPLEMENTARY TABLES

Supplementary Table 1. Clinical profile in GC patients in different age groups.

**Variables**	**Age <50 yrs n=222 n (%)**	**Age 50-70 yrs n=586 n (%)**	**Age >70 yrs n=941 n (%)**	***P* value**
Gender (M/F)	114/108	365/221	776/165	**<0.001**
Tumor size (<5/≥5 cm)	138/84	339/247	522/419	0.176
Cell differentiation				**<0.001**
Poor	186 (83.8)	327 (55.8)	367 (39.0)	
Moderate	35 (15.8)	250 (42.7)	555 (59.0)	
Well	1 (0.5)	9 (1.5)	19 (2.0)	
Gross appearance				**<0.001**
Superficial type	108 (48.6)	214 (36.7)	327 (34.8)	
Borrmann type 1 and 2	23 (10.4)	131 (22.5)	262 (27.9)	
Borrmann type 3 and 4	91 (41.0)	238 (40.8)	351 (37.3)	
Lauren's classification				**<0.001**
Intestinal type	54 (24.3)	302 (51.5)	666 (70.8)	
Diffuse type	168 (75.7)	284 (48.5)	275 (29.2)	
Lymphovascular invasion	99 (44.6)	300 (51.2)	513 (54.5)	**0.025**
Pathological T category				0.077
T1/2/3/4	81/26/93/22	209/84/202/91	320/158/319/144	
Pathological N category				0.465
N0/1/2/3	108/35/24/55	269/78/99/140	456/123/142/220	
Pathological TNM Stage				0.267
I/II/III	89/62/71	231/138/217	394/202/345	

TNM: Tumor, Node, Metastasis; Bold: statistically significant.

Supplementary Table 2. The initial recurrence pattern in gastric cancer patients in different age groups.

	**Age <50 yrs n=222 n (%)**	**Age 50-70 yrs n=586 n (%)**	**Age >70 yrs n=941 n (%)**	***P* value**
Total patients with recurrence	46 (20.7)	135 (23.0)	204 (21.7)	0.728
Locoregional recurrence	12 (5.4)	56 (9.6)	67 (7.1)	0.085
Distant metastasis	34 (15.3)	89 (15.2)	150 (15.9)	0.918
Peritoneal dissemination	22 (9.9)	44 (7.5)	59 (6.3)	0.153
Hematogenous metastasis	9 (4.1)	44 (7.5)	86 (9.1)	**0.037**
Liver	2 (0.9)	28 (4.8)	64 (6.8)	**0.002**
Lung	1 (0.5)	9 (1.5)	15 (1.6)	0.419
Bone	4 (1.8)	9 (1.5)	9 (1.0)	0.453
Brain	2 (0.9)	1 (0.2)	1 (0.1)	0.078
Adrenal	0	1 (0.2)	3 (0.3)	0.628
Skin	1 (0.5)	1 (0.2)	2 (0.2)	0.750
Distant lymphatic recurrence	5 (2.3)	20 (3.4)	36 (3.8)	0.513

Some patients had more than one recurrence pattern.

Supplementary Table 3. Multivariate analysis of factors affecting OS and DFS of GC patients after curative surgery.

Prognostic factors	OS			DFS		
	HR	95%CI	*P* value	HR	95%CI	*P* value
Age	1.67	1.495-1.865	**<0.002**	1.56	1.403-1.737	**<0.001**
Gender	0.81	0.688-0.953	**0.011**	0.81	0.690-0.950	**0.010**
Tumor size	1.25	1.082-1.452	**0.003**	1.23	1.067-1.422	**0.004**
Cell differentiation	1.04	0.854-1.256	0.720	1.03	0.849-1.238	0.795
Lymphovascular invasion	1.50	1.274-1.773	**<0.001**	1.51	1.285-1.776	**<0.001**
Lauren's classification	1.18	0.964-1.448	0.109	1.11	0.907-1.352	0.316
Pathological TNM stage	1.91	1.721-2.115	**<0.001**	1.94	1.754-2.145	**<0.001**

CI: confidence interval; HR: hazard ratio; OS: overall survival; DFS: disease-free survival. Bold: statistically significant; TNM: Tumor, Node, Metastasis; Bold: statistically significant.

Supplementary Table 4. Comparison of the molecular differences between different age groups.

Variables	Age <50 yrs<br>n=63<br>n (%)	Age 50-70 yrs<br>n=188<br>n (%)	Age >70 yrs<br>n=182<br>n (%)	*P* value
MSI status				**0.038**
MSI-H	1 (1.6)	18 (9.6)	23 (12.6)	
MSI-L/S	72 (98.4)	170 (90.4)	161 (87.4)	
HP infection	38 (60.3)	98 (52.1)	90 (49.5)	0.330
EBV infection	9 (14.3)	24 (12.8)	24 (13.2)	0.953
PIK3CA amplification	21 (33.3)	70 (37.2)	62 (34.1)	0.765
Genetic mutations				
PI3K/AKT pathway	10 (15.9)	20 (10.6)	27 (14.8)	0.387
TP53	6 (9.5)	25 (13.3)	16 (8.8)	0.354
ARID1A	2 (3.2)	14 (7.4)	20 (11.0)	**0.044**
BRAF	0	0	1 (0.5)	0.501

MSI: microsatellite instability; MSI-H: MSI-high; MSI-L/S: MSI-low/stable; HP: Helicobacter pylori; EBV: Epstein-Barr virus; Bold: statistically significant.
